# Using qualitative system dynamics modeling to understand overdose bystander behavior in the context of Connecticut’s Good Samaritan Laws and identify effective policy options

**DOI:** 10.1186/s12954-024-00990-3

**Published:** 2024-06-27

**Authors:** Rachel L. Thompson, Nasim S. Sabounchi, Syed Shayan Ali, Robert Heimer, Gail D’Onofrio, Rebekah Heckmann

**Affiliations:** 1 Center for Systems and Community Design, City University of New York Graduate School of Public Health and Health Policy, 55 West 125th Street, New York, NY 10027 USA; 2 Department of Health Policy and Management, City University of New York Graduate School of Public Health and Health Policy, 55 West 125th Street, New York, NY 10027 USA; 3 University of Pittsburgh Medical Center, University of Pittsburgh School of Medicine, 200 Lothrop Street, Pittsburgh, PA 15213 USA; 4 Department of Epidemiology of Microbial Diseases, Yale School of Public Health, 60 College Street, New Haven, CT 06520 USA; 5 Center for Interdisciplinary Research on AIDS at Yale, 135 College St., Suite 200, New Haven, CT 06520 USA; 6 Department of Emergency Medicine, Yale School of Medicine, 333 Cedar Street, New Haven, CT 065108 USA; 7 Department of Chronic Disease Epidemiology, Yale School of Public Health, 60 College Street, New Haven, CT 06520 USA

**Keywords:** Opioids, Opioid use disorder, Overdose, Harm reduction, System dynamics modeling, Systems science, Good Samaritan Laws

## Abstract

**Background:**

Good Samaritan Laws are a harm reduction policy intended to facilitate a reduction in fatal opioid overdoses by enabling bystanders, first responders, and health care providers to assist individuals experiencing an overdose without facing civil or criminal liability. However, Good Samaritan Laws may not be reaching their full impact in many communities due to a lack of knowledge of protections under these laws, distrust in law enforcement, and fear of legal consequences among potential bystanders. The purpose of this study was to develop a systems-level understanding of the factors influencing bystander responses to opioid overdose in the context of Connecticut’s Good Samaritan Laws and identify high-leverage policies for improving opioid-related outcomes and implementation of these laws in Connecticut (CT).

**Methods:**

We conducted six group model building (GMB) workshops that engaged a diverse set of participants with medical and community expertise and lived bystander experience. Through an iterative, stakeholder-engaged process, we developed, refined, and validated a qualitative system dynamics (SD) model in the form of a causal loop diagram (CLD).

**Results:**

Our resulting qualitative SD model captures our GMB participants’ collective understanding of the dynamics driving bystander behavior and other factors influencing the effectiveness of Good Samaritan Laws in the state of CT. In this model, we identified seven balancing (B) and eight reinforcing (R) feedback loops within four narrative domains: Narrative 1 - *Overdose, Calling 911, and First Responder Burnout*; Narrative 2 - *Naloxone Use, Acceptability, and Linking Patients to Services*; Narrative 3 - *Drug Arrests, Belief in Good Samaritan Laws, and Community Trust in Police*; and Narrative 4 - *Bystander Naloxone Use, Community Participation in Harm Reduction, and Cultural Change Towards Carrying Naloxone*.

**Conclusions:**

Our qualitative SD model brings a nuanced systems perspective to the literature on bystander behavior in the context of Good Samaritan Laws. Our model, grounded in local knowledge and experience, shows how the hypothesized non-linear interdependencies of the social, structural, and policy determinants of bystander behavior collectively form endogenous feedback loops that can be leveraged to design policies to advance and sustain systems change.

**Supplementary Information:**

The online version contains supplementary material available at 10.1186/s12954-024-00990-3.

## Background

Opioid use disorder (OUD) and fatal opioid overdose continue to pose significant and persistent burdens to human health and quality of life in the United States. Nationally, opioid overdose deaths continue to climb at an alarming rate. Since 1999, over 644,000 people have died from an overdose involving opioids; and, between 2019 and 2021, deaths increased by 61%, from 49,860 to 80,411 [1]. In Connecticut (CT), the age-adjusted rate of drug-induced mortality in 2020 was 39.1 deaths per 100,000, with 93% involving opioids -- 38% higher than the national rate of 28.3 deaths per 100,000 with 75% of deaths involving opioids [1–3]. Opioid use disorder (OUD) and fatal opioid overdose cost the U.S. an estimated $1 trillion and the state of CT $17.2 billion annually, which includes component costs of managing OUD (healthcare costs, substance use treatment, criminal justice, lost productivity, and reduced quality of life) and the healthcare costs, lost productivity costs, and value of life lost by fatal opioid overdose [4].

Good Samaritan Laws are a harm reduction policy intended to reduce fatal opioid overdose by protecting bystanders, first responders, and health care providers who assist individuals experiencing an overdose from civil or criminal liability. Presently, all fifty states and Washington DC have some form of Good Samaritan Law [5, 6]. In 2017, Canada enacted national legislation through the Good Samaritan Drug Overdose Act, becoming one of the first and only examples of sweeping, nationwide, drug-related Good Samaritan Laws [7, 8]. Most Good Samaritan Laws include some protection against arrest or criminal liability for bystanders and first responders who aid someone experiencing an overdose by calling 911 or administering naloxone; however, levels of immunity vary, and many states in the US do not offer protection to the individuals experiencing the overdose [5]. CT’s Good Samaritan Laws were originally implemented in 2011 and were extended and updated in 2012, 2014, and 2015 [9]. In their current iteration, the laws provide civil and criminal immunity to any bystander who provides or administers naloxone in the event of a suspected opioid overdose or seeks emergency care for themselves or any other person experiencing an overdose [10]. CT’s Good Samaritan Laws are relatively protective compared to other states in that they offer specific protections to the individual experiencing an overdose; however, these protections are not afforded when an arrest is being served or a search warrant is authorized [5].

Evidence demonstrating the effectiveness of Good Samaritan Law implementation at reducing overdose fatalities in the US and Canada has been mixed so far, with some studies showing between 10% and 15% lower incidence of opioid overdose deaths 1–2 years after enactment [11, 12] and other studies showing no significant association between Good Samaritan Laws and opioid overdose deaths [13, 14]. One reason why Good Samaritan Laws may not be achieving greater impact is that, in many communities, the protections offered by the laws are not fully understood by potential bystanders. Studies have shown that awareness and correct knowledge of locally applicable Good Samaritan Laws remains low among people who use drugs and potential bystanders [15–18]. Better knowledge of Good Samaritan Laws has generally been associated with an increased frequency of bystanders calling 911 and otherwise seeking medical help when witnessing an overdose [16, 19–21].However, even when knowledge about the laws is correct, distrust of law enforcement and fear of legal consequences may stand in the way of bystanders seeking help [18, 20].

The determinants of bystander behavior are complex, dynamic, and locally specific, requiring a systems perspective to fully understand and characterize. Systems science methods are increasingly being utilized to study public health problems, stemming from the growing recognition that public health problems are inherently complex and contain non-linear interdependencies that cannot be fully characterized by linear thinking and models [24, 25]. We recently developed a system dynamics (SD) simulation model to evaluate the impacts of CT’s Good Samaritan Laws on overdose deaths, emergency department visits for overdose, and behavioral changes of bystanders (e.g., calling 911 for overdose, using naloxone) [26]. In this study, we expand on that previous work to gain a greater qualitative and systems-level understanding of the numerous dynamic social, structural, and policy factors influencing bystander responses to opioid overdose in the context of CT’s Good Samaritan Laws.

## Methods

Group model building (GMB) is a participatory approach to SD modeling, which has been used to study a variety of complex social issues, including health disparities, structural racism, stigma, and opioid use [27–30]. Typical GMB programs bring together community members, experts, and other stakeholders to work together with modeling facilitators to build a shared understanding of the forces and feedback processes that explain a complex, dynamic problem [25, 31, 32]. SD modeling comprises a set of methodologies that are designed to facilitate understanding of the structure and behavior of complex systems and can be leveraged to design policies for effective and sustained systems change [33]. GMB exists within traditions of community-based participatory research and community-based system dynamics, whereby research is conducted as an equitable partnership between researchers and members of the community with outcomes that are designed to benefit the community [32, 34]. In our study, we conducted a series of six GMB workshops to elicit knowledge and experiences of local health care experts, harm reduction professionals, first responders, and community members with lived experience and to develop a shared understanding of the system influencing bystander behavior and the effectiveness of CT’s Good Samaritan Laws. Using this collective knowledge, we developed a qualitative system dynamics model and, as a group, explored the elements of the model to identify key policy levers with the potential to improve public health outcomes.

### Participant recruitment

Potential GMB workshop participants were recruited through existing networks and partnerships held by members of the research team at Yale University and collaborators at the Connecticut Department of Public Health. Individuals from across the state with experience and expertise relevant to the discussion of Good Samaritan Laws in CT were invited to GMB workshops via email solicitation. All GMB sessions were held virtually using video conferencing technology to allow for wider engagement of participants located across the state. To further maximize attendance, dates and times for workshop sessions were scheduled based on the availability of participants.

### GMB sessions 1, 2, & 3: preliminary modeling exercises

For our first three GMB workshops, held in March, April, and July 2021; we invited a diverse set of stakeholders with expertise and lived experience within the context of the opioid epidemic in CT. In March (session 1), we met with 13 experts in health care, public health, and harm reduction; in April (session 2), we met with 14 first responders, police officers, and firefighters; and in July (session 3) we met with 7 harm reduction experts and community members who had experienced or witnessed an opioid overdose. Participants were grouped together based on similar perspectives so that individuals would have the space to speak freely and openly share their thoughts, minimizing the impact of any power dynamics on the results of the session. The main goals of these sessions were to (1) identify key facilitators and barriers impacting the effectiveness of Good Samaritan Laws in CT, (2) obtain reference modes for these key factors by having participants construct Behavior over Time Graphs [35], and (3) create causal loop diagrams (CLDs) capturing feedback relationships that could potentially explain the key factors using direct input from participants.

During the three GMB sessions, participants were prompted to brainstorm and identify potential facilitators of and barriers to effectiveness of Good Samaritan Laws in CT. Participants were also asked to identify and explain factors influencing bystander behavior during an overdose, distribution of naloxone, and reduction of opioid overdose deaths in the state. We further engaged participants on these ideas using a Behavior over Time Graphs exercise [35], where participants were asked to draw out how they perceived these important factors to have changed over time. Participants were prompted to choose the Behavior over Time Graphs they thought were most important and discuss their rationale with the larger group. An initial CLD “seed model” was presented to each group and built upon by incorporating constructs and variable connections discussed by the participants in real time using Vensim ® DSS (https://vensim.com/) software. The seed model and a selection of Behavior over Time Graphs drawn by participants are available in the supplement (***S1*** and ***S2***, respectively). During each GMB session, the research team collected an extensive and detailed set of field notes, transcripts, and other cognitive artifacts that were subsequently analyzed and interpreted within the grounded theory framework of qualitative research [36, 37]. Constructs that emerged from the GMB sessions were mapped to qualitative SD models in the form of CLD structures, variables, and variable connections.

### GMB sessions 4 & 5: model follow-up & participant feedback

A second set of GMB workshops were held in October 2021 with a combination of new and previous participants. All previous participants were extended invitations to join these follow-up sessions, although some were not available, and some did not respond to the invitation. In each session, the goals were to (1) critically evaluate and refine qualitative SD model structures identified in the previous GMB sessions, (2) discuss policy scenarios identified in previous sessions, and (3) gain insights from participants as to how these policies might interface with our preliminary system feedback structures. In these sessions, individuals representing a variety of perspectives were included together to generate a richer critical discussion, and to encourage participants to consider other perspectives. In all, six first responders, harm reduction experts, and public health professionals attended the first session; and six harm reduction, public health, and health care professionals attended the second session.

During these sessions, we presented the CLDs resulting from each of the initial GMB sessions (session 1: health care providers, session 2: law enforcement & first responders, and session 3: harm reduction, bystanders, and individuals with lived experience of an opioid overdose) to participants for discussion and feedback. For each of the CLDs, we identified and walked through several feedback loops central to the narratives discussed by each of the original three stakeholder groups. Participants were asked a series of guided questions intended to facilitate critical interaction with the CLDs for validation of presented feedback structures and model constructs. Furthermore, we facilitated discussion on policy scenarios identified by participants in previous sessions for additional insights from the participants on the perceived intended and unintended impacts of implementing such policies in real life and in the context of our qualitative SD model.

CLDs produced during the first three GMB sessions were iteratively refined with participant input after presentation back to stakeholder groups in GMB sessions 4 & 5. After the first five GMB sessions, individual CLDs were merged by systematically consolidating like concepts from each CLD into single variables in a merged CLD. Key feedback loops in the merged model were identified by the project team, and a simplified CLD was presented to stakeholders in a final plenary GMB session for feedback and validation of the model (see next section).

### GMB session 6: policy agenda

All participants who previously attended at least one previous GMB session were invited to a final plenary GMB session in January 2022. Twelve harm reduction, public health, and health care professionals were able to attend the session. The primary aims of the plenary were to (1) present a synthesis of key findings and narratives from previous GMB efforts to the stakeholders for further comment and discussion and (2) identify the top policy priorities of the present stakeholders given the set of policies identified by participants in previous GMB sessions.

In this session, we introduced participants to a CLD resulting from the synthesis of constructs identified in individual GMB sessions and simplified to produce a set of feedback loops central to the narratives and themes that emerged from the previous five modeling workshops. We briefly walked through each of the feedback loops represented in the model and discussed the story behind each loop using systems thinking. We then conducted a policy agenda exercise in which we asked participants to rank the set of potential policies and interventions identified in the previous five GMB sessions, resulting in the identification of nine high-priority strategies with the greatest potential for public health impact in CT (see supplement ***S3*** for the full list of 64 policies identified). Finally, we discussed how the top selected policies might interact with feedback loops in our qualitative SD model and asked participants to share their perspectives on potential intended or unintended consequences of policy implementation.

### Qualitative system dynamics model (causal loop diagram)

Our final qualitative SD model, represented in the form of a CLD, resulted from a systematic, iterative process of synthesis, refinement, and simplification, grounded in participant knowledge elicited during GMB sessions. Background knowledge and expertise of the project team was utilized when necessary to support modeling decisions when field notes from participants were unclear or incomplete. A final set of four key feedback loop narratives reflecting stakeholder-identified policy priorities was selected for further discussion in this paper. The full version of our qualitative SD model is available as a supplemental Vensim® model file (***S4***).

The CLD presented in this paper follows standard SD modeling practices [33], containing variable names and arrow linkages with positive (+) and negative (-) signs. Arrow linkages in CLDs represent hypothesized causal relationships between variables. Positive arrows indicate that a change in the first variable produces a change in the same direction of the variable that follows (i.e., an increase in variable X produces a subsequent increase in variable Y). Negative arrows indicate that a change in the first variable produces a change in the opposite direction of the variable that follows (i.e., an increase in variable X produces a subsequent decrease in variable Y). A closed series of arrows represents a feedback loop which can either produce reinforcing or balancing effects on the system. Reinforcing (R) feedback loops contain an even number of negative linkages and tend to produce exponential growth or decline, driving change in the system. In contrast, balancing (B) feedback loops contain an odd number of negative linkages and tend to produce goal-seeking behavior, stabilizing the effects of disruptions to the system and bringing variables into steady states.

## Results

### Model overview

**Fig. 1:**
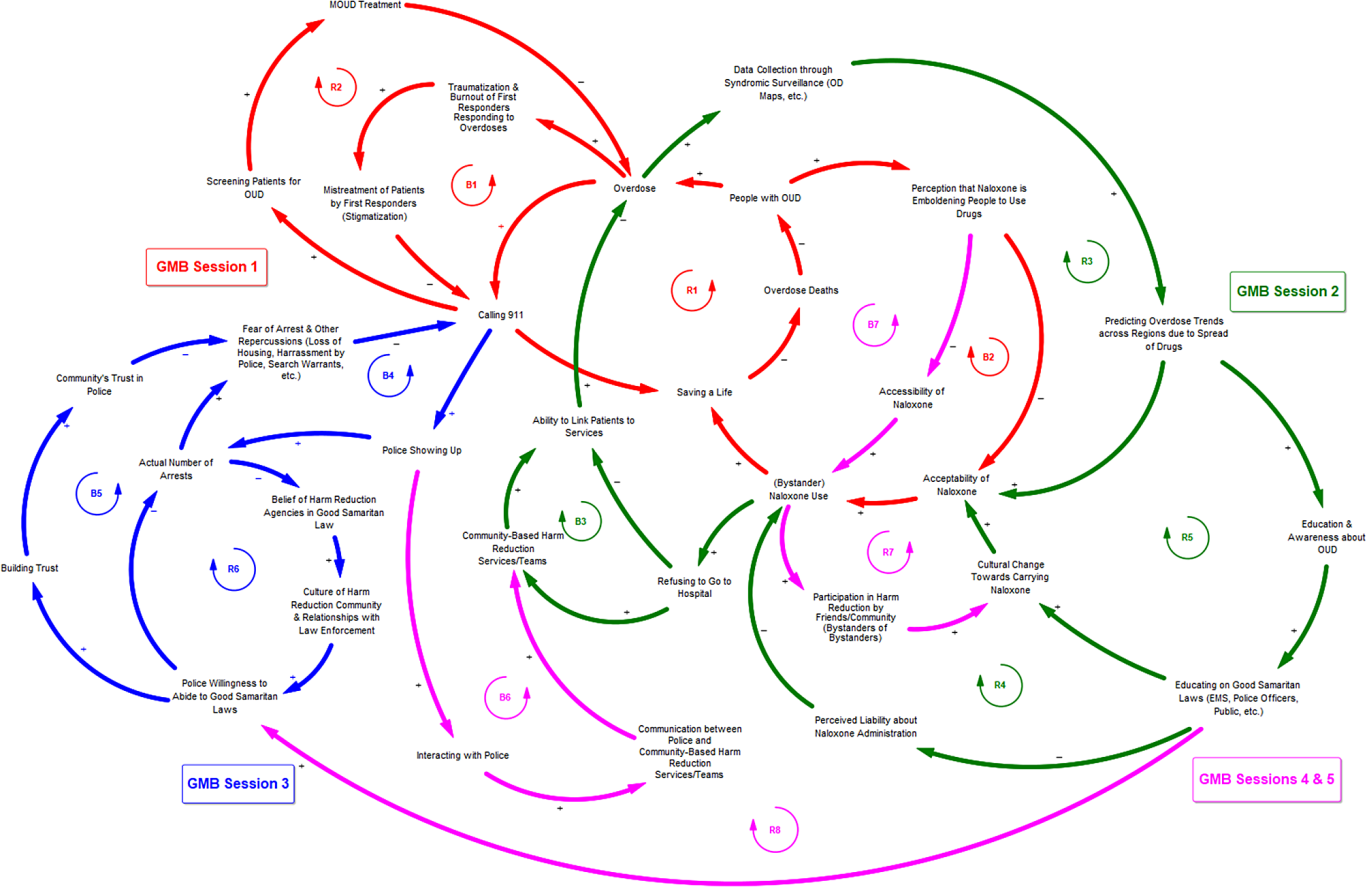
A qualitative system dynamics model of bystander behavior in the context of CT’s Good Samaritan Laws. This model is a synthesis and simplification of feedback loops identified during GMB session 1 (health care providers & harm reduction experts), GMB session 2 (law enforcement & first responders), GMB session 3 (harm reduction experts & individuals who have experienced or witnessed an overdose), and GMB sessions 4 & 5 (combined participant sessions)

The overview model presented in Fig. 1 contains four model subsections corresponding to key feedback loops identified during the GMB sessions. The loops shown in red represent input from healthcare providers & harm reduction experts in GMB session 1. The loops shown in green represent input from law enforcement & first responders in GMB session 2. The loops shown in blue represent input from harm reduction experts & individuals who have experienced or witnessed an overdose in GMB session 3. The loops shown in pink emerged from panels of combined participants convened for GMB sessions 4 and 5.

Selected feedback loops from GMB session 1 explain the process by which individuals who experience an overdose and call 911 may subsequently interact with the healthcare system, resulting in linkages to treatment (R1, R2). Other loops from this session focus on cycles of burnout and traumatization among first responders who respond to overdoses (B1) and community perceptions of naloxone use emboldening people to use drugs as a barrier to naloxone acceptability (B2). Selected loops from GMB session 2 highlight feedback processes centered on bystander naloxone use and accessibility of naloxone in the community (R3, B3, R4, R5). Some of these loops explain alternative linkages to care, such as through community-based harm reduction teams (B3); and some describe the impacts of education about Good Samaritan Laws on naloxone use and the processes of data utilization by public health departments and other organizations as drivers of education initiatives and changing community perceptions of the epidemic (R4, R5). Selected feedback loops from GMB session 3 focus on community interactions with and trust in law enforcement and how fear of arrest and other repercussions may discourage individuals from calling 911 in the event of an overdose (B4, B5, R6). These loops also highlight the important role of relationship building between police and community-based groups to encourage a culture of harm reduction and enhance community belief in Good Samaritan Laws and trust in police. Four additional loops added after review of notes and discussions from GMB sessions 4 and 5 provide greater context and interconnectedness of previously discussed system feedback structures. These additional loops describe feedback processes related to naloxone acceptance and use (R7, B7), interactions between police and community-based harm reduction teams (B6), and effects of education on police willingness to abide by Good Samaritan Laws (R8). Several of these loops and their interactions are described in greater detail in the following discussion on model narratives.

### Model narratives

#### Narrative 1 - Overdose, Calling 911, and First Responder Burnout (Fig. 2)

**Fig. 2:**
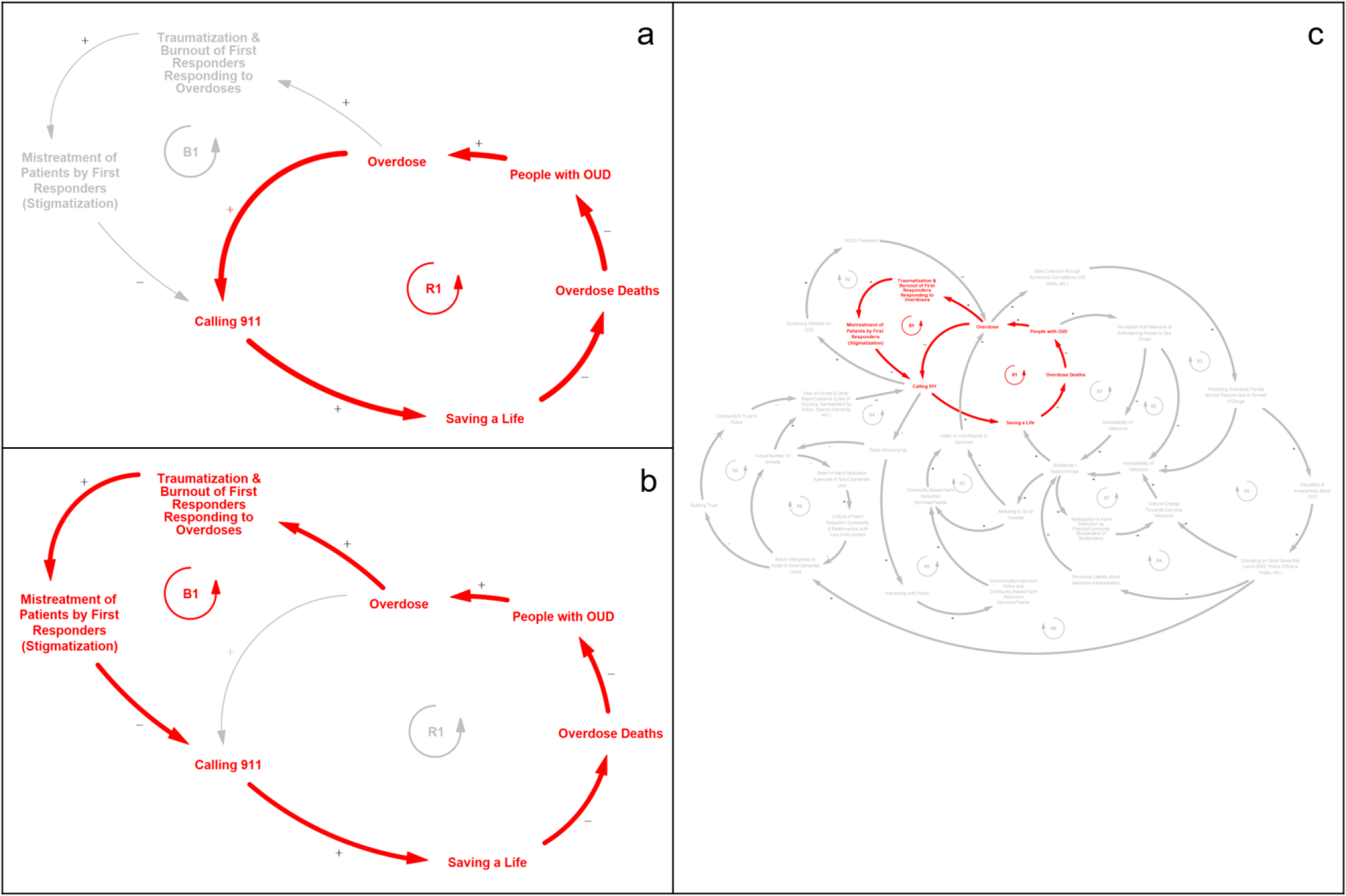
Feedback loops from model narrative 1 - *Overdose, Calling 911, and First Responder Burnout;* (a) reinforcing feedback loop R1; (b) balancing feedback loop B1; (c) R1 and B1 placement within larger CLD

Our first model narrative explains some of the mechanisms associated with interactions between people experiencing an overdose and first responders and is illustrated by two competing feedback loops, the reinforcing loop R1 (Fig. 2a) and the balancing loop B1 (Fig. 2b). R1 shows how calling 911 after an overdose can save lives which, in turn, sustains a higher population with OUD in the absence of linkages to treatment or other interventions aimed at reducing overdoses. As illustrated by R1, as there are more overdoses, an increased number of 911 calls are made to request that first responders attend to individuals experiencing an overdose; this leads to more lives saved and a reduction in deaths. Given that there is now a larger population of individuals living with OUD (independent of linkages of patients to treatment such as medication for opioid use disorder (MOUD)), there is now the potential for more overdoses and, thus, even more calls to 911 for assistance, perpetuating the reinforcing feedback cycle of overdoses in the community.

The competing balancing feedback loop B1 illustrates the impacts of first responder burnout on overdoses and overdose deaths, whereby treatment of patients that perpetuates stigma may lead to downstream effects of increased overdose deaths and decreased populations of people living with OUD in the community due to hesitation of bystanders to call 911. Loop B1 illustrates how an increase in overdoses in the community contributes to the traumatization and burnout of first responders attending to numerous overdoses, particularly repeated overdoses at the same address or with the same individual(s). Burnout of first responders may subsequently lead to mistreatment of individuals experiencing an overdose, contributing to increased stigma and negative attitudes towards people who use drugs. This stigmatized treatment may, in turn, discourage patients and bystanders from calling 911 for fear of mistreatment by first responders. Ultimately, this could lead to fewer lives saved by first responder action and an increase in deaths from overdose in the community. The unfortunate balancing effect of this cycle comes from the reality that, as more overdose deaths occur, there are fewer individuals with OUD alive, which reduces the number of overdoses to which first responders are summoned.

### Narrative 2 - Naloxone Use, Acceptability, and Linking Patients to Services (Fig. 3)

**Fig. 3:**
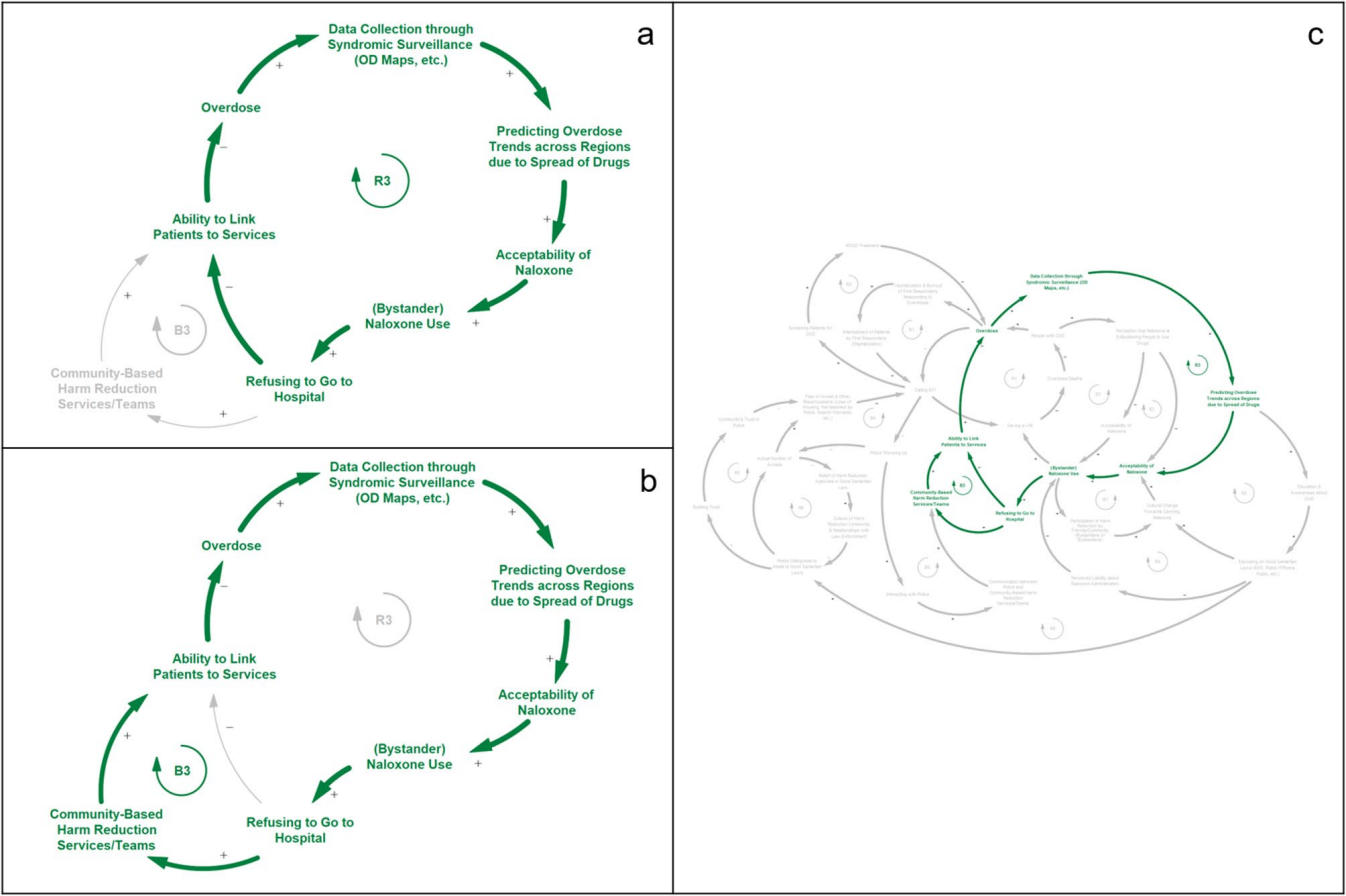
Feedback loops from model narrative 2 - *Naloxone Use, Acceptability, and Linking Patients to Services;* (a) reinforcing feedback loop R3; (b) balancing feedback loop B3; (c) R3 and B3 placement within larger CLD

The second model narrative is characterized by another set of competing feedback loops, reinforcing feedback loop R3 (Fig. 3a) and balancing feedback loop B3 (Fig. 3b). This narrative shows the interacting factors associated with the acceptance and use of naloxone by bystanders and the linkage of people who use drugs to treatment and social services. The narrative captured by the reinforcing feedback loop R3 shows how increased naloxone use, in the complete absence of linkages to treatment, may have the unintended downstream effect of an increased number of overdoses, necessitating further use of naloxone. When overdoses rise in a community, syndromic surveillance systems such as OD maps capture the rise in overdoses, leading to further public health efforts to predict overdose trends. These heightened surveillance activities and resulting public health communications to the community can produce a subsequent increase in the acceptability of naloxone due to public awareness of the rising opioid problem in their community. As public acceptability of naloxone increases, bystander naloxone use subsequently rises, leading to fewer individuals who experience overdose going to the hospital. If fewer overdose victims interact with the healthcare system following successful bystander administration of naloxone and are not linked to treatment, this may perpetuate rising overdoses in the community and demand for bystander naloxone use.

The balancing feedback loop B3 provides a direct counteraction to the reinforcing feedback cycle described in R3. In this loop, individuals refusing to go to the hospital following the successful administration of naloxone may be linked to services through community-based harm reduction services instead of through a hospital-based healthcare system. Like the process in R3, when overdoses rise in a given community, public health officials can predict overdose trends with more accuracy and alert the community by tapping into syndromic surveillance and other data sources for early detection of overdose spikes. Public health attention to the problem leads to greater acceptance and use of naloxone by the community. In contrast to R3, however, B3 depicts a process in which overdoses in the community stabilize as a result of overdose victims being linked to treatment and other services through the presence of community-based harm reduction teams.

### Narrative 3 - Drug Arrests, Belief in Good Samaritan Laws, and Community Trust in Police (Fig. 4)

**Fig. 4:**
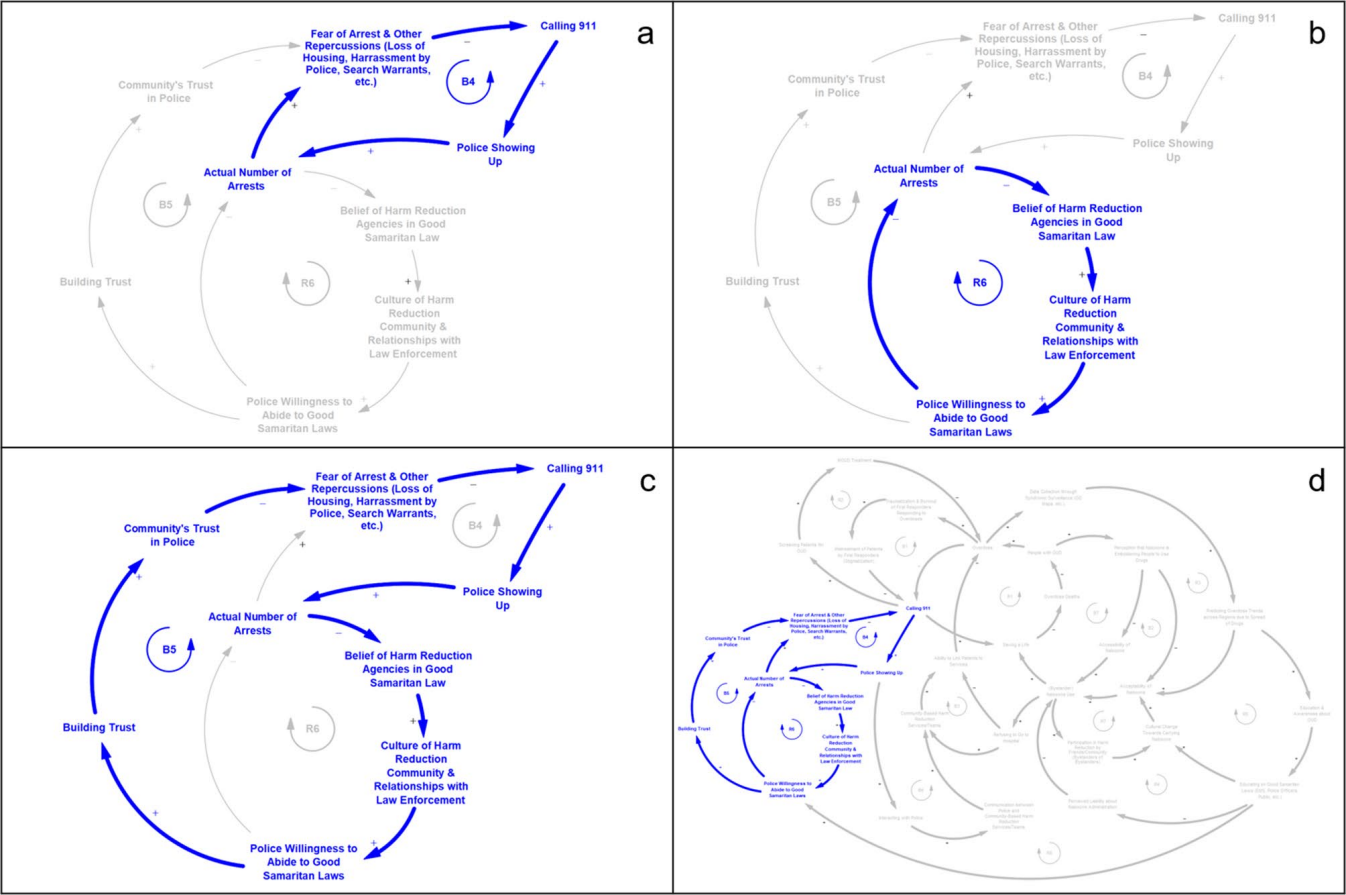
Feedback loops from model narrative 3 - *Drug Arrests, Belief in Good Samaritan Laws, and Community Trust in Police*; (a) balancing feedback loop B4; (b) reinforcing feedback loop R6; (c) balancing feedback loop B5; (d) B4, R6, and B5 placement in larger CLD

The third model narrative, described by a set of three interacting feedback loops—balancing feedback loops B4 and B5 (Fig. 4a and c) and reinforcing feedback loop R6 (Fig. 4b)—illustrates how communities interact with and perceive police and how these interactions and perceptions affect utilization of 911 during an overdose. The balancing feedback process B4 describes the situation in which interactions of individuals who use drugs with police promote avoidance of seeking help through 911 for fear of arrest and other repercussions from law enforcement. When individuals who witness an overdose call 911, there is a chance that police may show up at the scene of the overdose. There is, consequently, an increased likelihood that a drug arrest may occur, as opposed to scenarios where only EMS or firefighters arrive at the scene. Fear of arrest and other repercussions such as loss of housing or custody of children when police arrive at the scene may lead bystanders and past overdose victims to avoid calling 911 in the event of future overdoses, which can have other downstream system effects (e.g., increased risk of death, decreased linkages to treatment and other services).

R6 describes a reinforcing feedback cycle by which a culture of harm reduction within law enforcement and the community builds trust and positive relationships. As relationships improve, police become more willing to abide by Good Samaritan Laws, thereby leading to fewer drug arrests that violate the intention of the laws. This reduction has the potential to improve harm reduction agencies’ belief in the willingness of law enforcement agencies to follow Good Samaritan Laws, which, in turn, further improves the culture of harm reduction and relationships between harm reduction and law enforcement by building trust between the two groups.

The narrative described by balancing feedback loop B5 incorporates constructs from both loops R6 and B4, connecting concepts related to the quality of relationships between harm reduction and law enforcement to police willingness to abide by Good Samaritan Laws, increased community trust in police, and willingness of bystanders to call 911 for overdose assistance. When police show up in response to a 911 call for an overdose and arrest either the overdose victim or others involved, this contributes to a loss of belief among local harm reduction agencies that the Good Samaritan Laws are effective. This erodes the culture of harm reduction, damages relationships between law enforcement and the harm reduction community, and can further decrease police’s willingness to abide by the laws. The impact extends to the community, resulting in a decreased willingness of bystanders and previous overdose victims to call 911.

### Narrative 4 - Bystander Naloxone Use, Community Participation in Harm Reduction, and Cultural Change Towards Carrying Naloxone (Fig. 5)

**Fig. 5:**
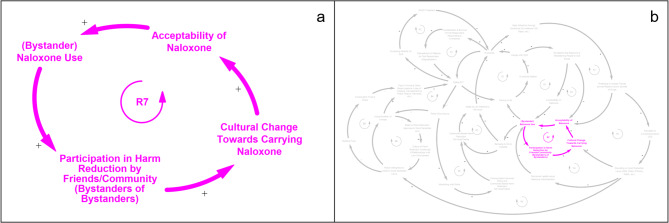
Feedback loops from model narrative 4 - *Bystander Naloxone Use, Community Participation in Harm Reduction, and Cultural Change Towards Carrying Naloxone* (a) reinforcing feedback loop R7; (b) R7 placement within larger CLD

The final model narrative is embodied by a single reinforcing feedback loop, R7 (Fig. 5a), that shows how cultural change towards carrying and using naloxone is driven by community participation in harm reduction. When naloxone is successfully administered by a bystander to someone experiencing an overdose, this may empower that individual and other witnesses to engage in harm reduction activities such as obtaining naloxone themselves and being trained in how to administer it. As more members of the community choose to become active participants in harm reduction efforts, the acceptability of naloxone use in the community is reinforced.

### Policy agenda

In the final plenary GMB session, we engaged participants in a policy agenda exercise to identify high-leverage policies that interface with our qualitative SD model and discuss the potential intended and unintended consequences of these policies using a feedback perspective. Out of a total of 64 policies and interventions identified by participants during the five GMB sessions (supplement ***S3***), stakeholders present at the plenary session identified nine that they collectively believed would have the greatest potential for curbing the opioid epidemic in CT (Table 1). We distilled these high-impact strategies into four broad policy themes based on the similarity of their impact on the system and corresponding feedback loops: (1) *naloxone access & use*, (2) *community-based harm reduction services & teams*, (3) *safer drug use*, and (4) *education to reduce stigma.* A list of the specific high-impact strategies and their corresponding policy theme is given in Table 1, along with a list of feedback loops and model narratives impacted by each specific strategy.

**Table 1:** Stakeholder-identified policy themes and selected high-impact strategies to improve opioid-related outcomes and effectiveness of CT’s Good Samaritan Laws, with interfacing model narratives and feedback loops

Policy Theme	Selected Strategy	Narratives	Feedback Loops
*Naloxone Access & Use*	● “Leave behind” program (i.e., leaving naloxone at the scene of an overdose)	2, 4	B2, B3, B7, R3, R4, R5, R7
	● EMS, fire, & police carrying and administering naloxone	1	B1, B2, B7, R1, R4, R5, R8
*Community-Based Harm Reduction Services & Teams*	● Connecting patients with addiction treatment services as quickly as possible after an overdose	2	B3, B6, R3
	● Recovery navigator program (i.e., pairing someone who has experienced an overdose with a first responder or patient advocate to link patients to services)	2	B3, B6, R3
	● Receiving addiction/social services at the site of an overdose	2	B3, B6, R3
*Safer Drug Use*	● Safe spaces to use	1	B1, B2, B7, R1, R4, R5, R8
	● A smartphone application that alerts others when the application user overdoses	1, 2, 3	B1, B3, B4, B5, B6, R1, R2, R3, R8
*Education to Reduce Stigma*	● Education of new law enforcement & emergency department staff, specifically to reduce stigma & poor treatment of patients	1, 3	B1, B4, B5, B6, R1, R2, R8
	● Engaging new medical trainees to change clinical culture	2, 4	B2, B3, B7, R2, R3, R4, R5, R7

## Discussion

Our final resulting qualitative SD model reflects our GMB participants’ collective understanding of the dynamic system driving overdose bystander behavior and other factors influencing the effectiveness of Good Samaritan Laws in the state of CT. This model as presented is a product of the GMB process reflecting the mental models of individuals present during the sessions. In the results section, we discussed the causal hypotheses captured by the CLD, although it is important to note that the causal relationships discussed have not been systematically evaluated and represent hypotheses only. Furthermore, the model presented in Fig. 1 is a simplified version of the full CLD obtained after synthesizing themes, variables, and relationships from all GMB sessions. This simplified version is presented so that the feedback loops and key insights most relevant to policy decision-making can be more easily described and understood.

Policy agenda themes and specific high-impact strategies identified by GMB participants in light of our qualitative SD model are generally well supported in the literature as effective evidence-based strategies to reduce opioid overdose mortality and OUD prevalence. Overdose education, naloxone distribution, and take-home naloxone programs are widely recognized as key evidence-based strategies for increasing public naloxone access and use, as well as improving awareness and reducing stigma around naloxone use [38–41]. Empirical studies have repeatedly demonstrated the effectiveness of these programs in preventing opioid overdoses and saving lives [39–43]. EMS-based naloxone “leave behind” programs and scaling up naloxone education and utilization among police, fire, and EMS broadly could provide opportunities for further expanding overdose education and naloxone distribution and take-home naloxone programs as effective means of reducing opioid overdose fatalities in CT and communities across the US [44–46].

Several recent initiatives have reported successes in leveraging community-based harm reduction services and teams to connect individuals who experienced an overdose to addiction treatment services, harm reduction, and social supports. In Massachusetts, the implementation of community-based post-overdose outreach programs linking opioid overdose survivors to addiction treatment and harm reduction services was associated with significantly lower opioid overdose fatality rates over time [47]. In CT, the implementation of an innovative protocol of substance use screening and linkage to treatment within EDs (Project ASSERT (Alcohol and Substance Abuse Services Education and Referral to Treatment)) has proven to be an effective means of linking individuals evaluated in the ED for substance use-related problems (including overdose) to evidence-based treatment services, such as immediate treatment with buprenorphine for patients who wish to initiate MOUD [48].

Safe drug consumption sites have historically been a controversial harm reduction approach with limited adoption in the US [49]. However, in other countries, implementation of safe consumption sites has been associated with reduced fatal and non-fatal overdoses and increased access to harm reduction services [49–51]. In North America, Canada has been pioneering the implementation of medically supervised safe drug consumption sites, with facilities operating in nearly every province, serving over 300,000 unique clients on a monthly basis [52]. In the two years following the implementation of the first safe drug consumption facility in Vancouver in 2003, fatal overdoses decreased by 35% in nearby city blocks and 9.3% across the city [50]. The opening of the first safe drug consumption facility in Vancouver was also associated with improved public order through reduced public injection drug use and syringe disposal [53].

Another novel approach for safer drug use as identified by our GMB participants – a smartphone application enabling individuals who use opioids to signal and respond to overdoses – has demonstrated promise for reducing fatal overdoses in preliminary studies [54]. In an observational study of volunteers using the smartphone app UnityPhilly in Philadelphia, use of the app led to successful overdose reversal in 95.9% of true overdose events; and intervention by laypersons preceded EMS response by 5 min or more in over half of overdose events [54]. Restricted access to harm reduction services and physical spaces for people who use drugs to use them together during the COVID-19 pandemic has also spurred digital health innovations to promote safer drug use [55, 56]. While not a new practice among people who use drugs, remote spotting (using a phone or video conference to witness someone using drugs and respond if an overdose occurs) has come to the forefront of discussion around leveraging digital tools to reduce overdose fatalities by avoiding solo drug use [55, 56]. Evidence from qualitative studies has shown that both informal spotting (provided by friends and family) and formal spotting services (provided by an organization, such as Never Use Alone https://neverusealone.com/) have the potential to mitigate harms associated with the opioid crisis, including increased safety of people who use drugs and reduced risk of overdose and death [57].

Finally, stigmatization of opioid use within medical and law enforcement institutions has been widely recognized as a barrier to adequate treatment services and harm reduction resources [58, 59]. Several interventions such as those proposed by our GMB participants have been implemented among nursing, medical, and pharmacy students and have demonstrated success in reducing stigmatizing attitudes through educational trainings, critical reflection, and/or direct contact with individuals with substance use disorders [60–65]. Among police officers, collaborative crisis intervention team trainings with mental health professionals are a promising avenue for reducing stigmatizing attitudes towards individuals with mental health and substance use disorders [66]. However, there remains a gap in understanding to what extent these interventions might affect access to and utilization of MOUD and harm reduction services by individuals with OUD. Insights from our GMB and resulting SD model indicate that targeted training of incoming medical and law enforcement staff could be a promising high-leverage area for future policy given that opportunities for impact through education are embedded within multiple interacting feedback loops affecting harm reduction uptake, relationships between medical and law enforcement institutions and the public, and utilization of emergency and addiction treatment services.

CT ranks in the top 10 among states with the highest rates of opioid-related overdose deaths in the nation [1], and the number of overdose deaths experienced in CT increased by 327% from 2012 to 2021 [67]. This problem has grown with the proliferation of deadly synthetic drugs like fentanyl [68], such that drug overdose is now considered a leading cause of fatal unintentional injury [69]. The identification of an interacting set of reinforcing and balancing feedback loops, consistent with findings from our previously published SD model [26], suggests that Good Samaritan Laws alone cannot effectively reduce the number of opioid-related deaths. Tackling the opioid crisis in the state of CT and beyond will require the design and implementation of innovative solutions that enhance the effectiveness of multiple harm reduction policies that target novel intervention points within the broader system. We believe that opioid policy design and implementation can be greatly enhanced using systems science tools such as GMB and SD.

Insights from our SD model suggest that implementation of existing policies in CT may be creating unintended barriers to reducing overdose deaths. Low awareness of the Good Samaritan Laws among people who use drugs who are in a position to witness and respond to an overdose limits the benefits of the Good Samaritan Law [15–18]. Furthermore, negative experiences as a bystander responding to an overdose, such as harassment by law enforcement, can also diminish enthusiasm for responding to an overdose. Negative experiences with law enforcement can spread among people who use drugs, leading to loss of faith in Good Samaritan Laws as expressed by some communities.

Our SD model identifies a need for expanded naloxone access in CT, but the rollout of publicly accessible naloxone vending machines has been complicated by state regulations. For example, CT state law dictates that naloxone vending machines located outdoors (and therefore available 24/7) be temperature-regulated in accordance with commercial storage suggestions [70]. However, laboratory experiments have shown that even extreme temperatures far above or below the suggested storage limits do not reduce the potency of naloxone [71].

Public health policies in the state may also be inadvertently creating competing incentives that reduce the effectiveness of Good Samaritan Laws. In response to rising deaths from accidental ingestion of opioids among young children in CT, the Connecticut Department of Children and Families has strengthened fentanyl testing policies for parents acting as caretakers of young children under the agency’s purview [72]. Increasing emphasis on surveillance of fentanyl use may have the unintended consequence of making parents involved in child welfare who use drugs more hesitant to call 911 in the event of an overdose for fear of repercussions by the state. As shown in our model, this can have further downstream consequences, including increased risk of overdose death and decreased linkage to harm reduction and addiction treatment services.

On a positive note, building collaborations across professional cultures (e.g., police, first responders, healthcare professionals, harm reduction experts) has been ongoing in several parts of CT. Most notable are the efforts of the Opioid Action Team in New London, CT, which is a multi-sectoral group of community organizations, treatment providers, government agencies, first responders, and community members living with substance use disorder who coordinate resources and efforts to respond to the opioid epidemic [73]. The New London Opioid Action Team reports that they are actively implementing interventions within their community to reduce stigma, coordinate access to treatment and recovery support services (including the deployment of “Recovery Navigators”) and achieve wide availability of naloxone [73]. Some members of the New London Opioid Action Team participated in our GMB sessions, which helped contribute to a common understanding of barriers to effectiveness of Good Samaritan Laws within their community. We have also heard that participation in the GMB project has helped enhance their efforts to expand naloxone access through directed distribution and public access to naloxone resupply boxes and increase uptake of buprenorphine prescribing within the New London community.

This study has some important limitations. Although we were able to effectively engage a large and diverse set of participants representing several important areas of expertise (e.g., harm reduction experts, health care professionals, first responders), we interacted with a limited number of individuals with lived experience of opioid use and overdose. We believe that the voices of these individuals are critical to understanding the social and structural context of bystander behavior and the unseen consequences of interventions targeted at people who use drugs. Furthermore, given our time and resource constraints, we were limited to completing only six GMB sessions. It is possible that, throughout these six sessions, theoretical saturation of qualitative insights was not achieved. However, there were several examples of similar narratives emerging during different GMB sessions with different sets of stakeholders which allowed us to merge CLDs created during independent sessions to obtain the final CLD presented in this paper. Finally, we acknowledge that our results may have been different with a different set of participants, which may have led to the discovery of different feedback structures as well as the identification of different policy priorities. Different combinations of participants at individual sessions may have also affected the results we obtained, although we did take steps to minimize the impact of power dynamics on our results by separating stakeholder groups in the initial exploratory GMB sessions (i.e., inviting first responders (police officers and fire fighters), healthcare/public health/harm reduction experts, and individuals with lived experience of overdose to different sessions).

Despite the limitations highlighted above, we believe this work makes a significant, novel contribution to the literature on bystander behavior in the context of Good Samaritan Laws. To our knowledge, we are the first team to use participatory systems science methods to facilitate a more realistic understanding of the complex, non-linear interdependencies of the social, structural, and policy determinants of bystander behavior. Throughout this study, we employed an iterative process of model development, validation, and revision centered around the voices of our diverse set of participants with a wide range of combined expertise, experience, and local knowledge. This allowed us to advance a shared understanding of the system and remain true to the nature of participatory research through partnerships between community and academic experts.

## Conclusion

Throughout six GMB sessions that engaged stakeholders with local knowledge and expertise, we used a participatory systems science approach to develop a qualitative SD model that gave us insights into the endogenous feedback processes governing bystander behavior in CT. This model elicited consensus around four high-leverage policies for improving opioid-related outcomes and effectiveness of Good Samaritan Law implementation in CT: (1) improving naloxone access and use, (2) scaling up community-based harm reduction services and teams, (3) enabling safer drug use, and (4) educating first responders and healthcare workers to reduce stigma.

### Electronic supplementary material

Below is the link to the electronic supplementary material.

Supplementary Material 1–3

Supplementary Material 4

## Data Availability

No datasets were generated or analysed during the current study.

## Abbreviations

OUD: opioid use disorder
CT: Connecticut
SD: system dynamics
GMB: group model building
CLD: causal loop diagram
R: reinforcing feedback loop
B: balancing feedback loop
MOUD: medication for opioid use disorder
