# Supplementary material for: Using qualitative system dynamics modeling to understand overdose bystander behavior in the context of Connecticut’s Good Samaritan Laws and identify effective policy options
Source: Harm Reduct J. 2024 Jun 27;21:124. doi: 10.1186/s12954-024-00990-3 (PMC11210010; doi:10.1186/s12954-024-00990-3)
Supplement: Supplementary file 2 — Supplementary Material 4 [file 12954_2024_990_MOESM2_ESM.docx]

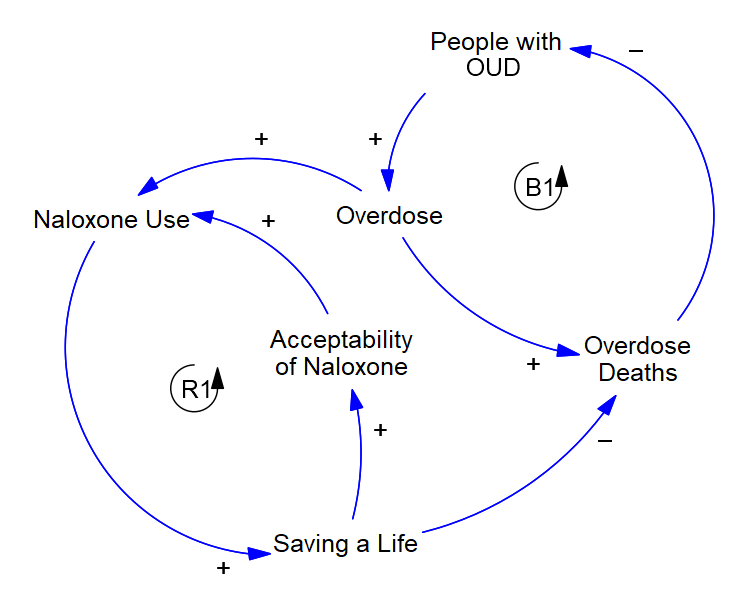


**S1)** “Seed model” causal loop diagram

| _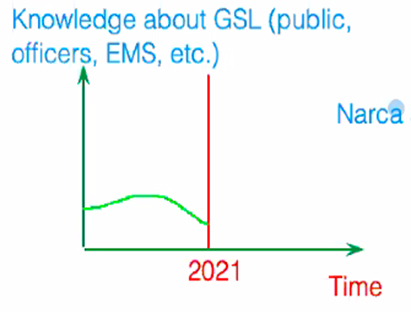_ | 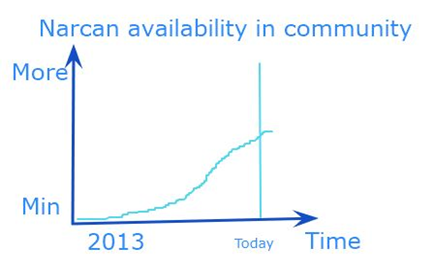  b  a | 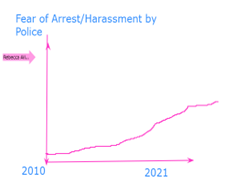  c |
| --- | --- | --- |

**S2)** Example behavior over time graphs constructed by group model building participants

| **Policy Area** | **Strategy** | **Ranking** |
| --- | --- | --- |
| *Harm Reduction – Naloxone Strategies* | - “Leave behind” program (leaving naloxone at the scene of an overdose) | 1^st^ |
|  | - EMS, fire, & police carrying and administering naloxone | 2^nd^ |
|  | - Reduce cost of naloxone | 3^rd^ |
|  | - Offering naloxone to individuals receiving medication for opioid use disorder (MOUD) | 4^th^ |
|  | - Increase local supply of naloxone | 5^th^ |
|  | - Targeted mailing of naloxone to high-risk persons (recently incarcerated, OD hot spots, etc.) | 6^th^ (tie) |
|  | - Increased availability of nasal naloxone | 6^th^ (tie) |
|  | - Pairing naloxone with defibrillators or placing naloxone in other publicly accessible locations | 8^th^ |
|  | - State policies that allow mailing of naloxone | 9^th^ |
|  | - Clinicians offering and prescribing naloxone | 10^th^ |
|  | - Automatically coupled electronic health record order of naloxone with opioid prescription | 11^th^ |
|  | - Screening providers about their feelings regarding naloxone prescribing (for targeted education) | 12^th^ |
| *Harm Reduction – Relationship Building Strategies* | - Community-based harm reduction services/teams | 1^st^ |
|  | - Following up with patients with addiction treatment services as quickly as possible after an overdose | 2^nd^ |
|  | - Rover program (harm reduction team handing out free naloxone in hot spots and building rapport in community) | 3^rd^ |
|  | - Building relationships between police and other agencies/community members | 4^th^ |
|  | - Receipt of social work services in the emergency department | 5^th^ |
|  | - Community-based peer education (e.g., sharing of experiences with naloxone) | 6^th^ (tie) |
|  | - Partnership between police & key people/decision-makers in the community | 6^th^ (tie) |
|  | - Police peer education and relationships (police sharing their experiences with other police about engaging with harm reduction) | 8^th^ |
|  | - 911 superusers (getting people who are frequent users of 911 system into treatment) | 9^th^ |
| *Harm Reduction – Other Strategies* | - Programs & agencies officially adopting harm reduction protocols & efforts | 1^st^ |
|  | - Safe spaces to use | 2^nd^ |
|  | - Safe supply of legal drugs | 3^rd^ |
|  | - Promoting use of the “buddy system” (not using alone) | 4^th^ |
|  | - Increase user awareness of fentanyl & other drug contamination | 5^th^ |
|  | - Participation in harm reduction by members of the community | 6^th^ |
|  | - Availability of drug contamination testing (e.g., fentanyl test strips) | 7^th^ |
|  | - Program for syringe disposal | 8^th^ |
| *Treatment Strategies* | - Recovery navigator program (pairing someone who has experienced an overdose with a first responder in order to link patients to services) | 1^st^ |
|  | - Receiving addiction/social services at the site of overdose | 2^nd^ |
|  | - Facilitating transportation of patients to treatment | 3^rd^ |
|  | - Syringe service programs initiating medication for OUD (MOUD) | 4^th^ (tie) |
|  | - EMS initiating medication for OUD (MOUD) | 4^th^ (tie) |
|  | - Hospital-based harm reduction services/teams | 6^th^ |
|  | - Dedicated hospital-based addiction services team | 7^th^ |
|  | - Additional/novel medication for treatment of OUD | 8^th^ |
| *Data Utilization Strategies* | - Utilizing OD maps data/SWORD/EMS dispatch information to track overdose hot spots | 1^st^ |
|  | - App that alerts others when the app user overdoses | 2^nd^ |
|  | - Data sharing with local public health department | 3^rd^ |
|  | - App integrating overdose data and community alerts/linkages | 4^th^ |
|  | - Updated app/website with clear & up-to-date guidelines of current Good Samaritan Law provisions for use by professionals | 5^th^ |
| *Education & Media Utilization Strategies* | - Education of law enforcement & emergency department staff, specifically to reduce stigma & poor treatment of patients | 1^st^ |
|  | - Engaging new medical trainees to change clinical culture | 2^nd^ |
|  | - Education through social media (e.g., awareness of fentanyl) | 3^rd^ |
|  | - Educating community on naloxone use & training on administration | 4^th^ |
|  | - Education of patients about medication for OUD (MOUD) | 5^th^ |
|  | - Utilizing social media for promoting support and peer navigation | 6^th^ |
|  | - Educating public on naloxone locations in community | 7^th^ (tie) |
|  | - Education of general public on opioid use disorder | 7^th^ (tie) |
|  | - TV/radio commercials for naloxone & Good Samaritan Laws | 9^th^ |
|  | - Education of providers about MOUD | 10^th^ |
|  | - Ads for naloxone in the community/police stations | 11^th^ |
|  | - Detailed flyers as a communication tool about Good Samaritan Laws | 12^th^ |
|  | - General education on Good Samaritan Laws (EMS, police officers, public, private businesses, etc.) | 13^th^ |
|  | - Education public on dangers of drug use | 14^th^ |
|  | - Getting more people involved and engaged in Good Samaritan Laws | 15^th^ |
| *Legal Strategies* | - Health insurance increasing access to medication for opioid use disorder (MOUD) | 1^st^ |
|  | - Address gaps in protection from Good Samaritan Laws (e.g., potentially losing custody of a child or housing, despite Good Samaritan Law protections) | 2^nd^ |
|  | - Specific liability protections in Good Samaritan Laws for businesses | 3^rd^ |
|  | - Mandatory transport to hospital after overdose only for high-risk patients | 4^th^ |
|  | - Mandatory transport to hospital after overdose | 5^th^ |
|  | - 72-hour hold (mandatory hold initiated at scene of overdose) for high-risk patients only | 6^th^ |
|  | - 72-hour hold (mandatory hold initiated at scene of overdose) | 7^th^ |

**S3)** Complete list of policies and interventions identified during group model building sessions. Participants present at the plenary group model building session ranked the strategies in seven policy areas in order from greatest potential impact to least potential impact assuming no limit to funds and resources.
